# ‘It’s common sense that an individual must eat’: Advocating for food justice with people with psychiatric disabilities through photovoice

**DOI:** 10.1111/hex.13101

**Published:** 2020-07-15

**Authors:** Lara Carson Weinstein, Mariana Chilton, Renee Turchi, Ann C Klassen, Marianna LaNoue, Alexis Silvero, Leopoldo J Cabassa

**Affiliations:** ^1^ Department of Family and Community Medicine Sidney Kimmel Medical College Thomas Jefferson University Philadelphia PA USA; ^2^ Dornsife School of Public Health, Health Management and Policy Drexel University Philadelphia PA USA; ^3^ Dornsife School of Public Health Community Health and Prevention Drexel University Philadelphia PA USA; ^4^ School of Population Health Thomas Jefferson University Philadelphia PA USA; ^5^ Brown School of Social Work Washington University in St. Louis St. Louis MI USA

**Keywords:** health and human rights, health disparities, obesity, serious mental illness

## Abstract

**Background:**

People with SMI have often been excluded in advocacy efforts focused on physical health, health care and health and social policy.

**Objective:**

Following a Photovoice project focused on barriers to healthy eating and physical activity in urban neighbourhoods, participant‐researchers were invited to present their insights in community advocacy settings. The purpose of this study was to explore the feasibility and participant–researchers’ experience of these community advocacy activities.

**Design:**

We held four focus groups with the eight participant‐researchers after each community advocacy activity to explore their experience with public speaking, presenting their experiences and advocating.

**Setting and Participants:**

People with serious mental illness who were overweight/obese living in supportive housing.

**Analysis approach:**

Qualitative analysis of the focus group transcripts, using a modified grounded theory approach followed by structured coding focused on empowerment, participation and non‐discrimination.

**Results:**

Participant‐researchers gave three oral presentations of their photographs at a variety of community‐based programmes and settings and participated in a rally to advocate for SNAP benefits. Two themes emerged from analysis: (a) Empowerment (the level of choice, influence and control that users of mental health services can exercise over events in their lives) and (b) Barriers to Empowerment (obstacles to participation and well‐being).

**Conclusions:**

This evaluation strengthens the evidence that it is feasible for participant‐researchers in Photovoice projects to engage in robust advocacy activities, such as presentations and discussions with local policymakers. During focus groups, participant‐researchers demonstrated realistic optimism towards their roles as change agents and influencers in spite of acknowledged systemic barriers.

## 1 INTRODUCTION

People with serious mental illness (SMI) experience significantly worse health than the general US population, a situation that is compounded all too often by homelessness and poverty.^1^, ^2^, ^3^ Community inclusion, or the freedom and opportunity to participate fully in society, is now recognized as a critical component of both mental health recovery and physical health in people with SMI.^3^ Policies and initiatives such as the American with Disabilities Act the President's New Freedom Commission on Mental Health and supportive housing for people with disabilities experiencing homelessness have made the pursuit of a healthy and meaningful life in the community a real possibility for people with SMI.^4^, ^5^, ^6^, ^7^ Self‐advocacy skills for people with SMI living in supportive housing are a key component of programmes such as Wellness Recovery Action Planning,^8^ and self‐advocacy efforts have demonstrated positive effects on hopefulness and quality of life in people with SMI.^9^

Despite recent advances, people with SMI have traditionally been excluded from larger advocacy efforts for physical health, health care and health and social policy. The discourse on efforts to address poor physical health of people with SMI historically focused almost exclusively on individual level factors, such as recommendations for dietary modification. This approach often ignores the influence of the social determinants of health (SDH), including homelessness and unstable housing, particularly in people with disabilities. The condition of food insecurity is defined by the United States Department of Agriculture as ‘household‐level economic and social condition of limited or uncertain access to adequate food’.^10^ An analysis by Coleman‐Jensen & Nord found that a person with a disability would require more than two‐and‐a‐half times the income of a person without disabilities to have the same likelihood of food security.^11^ Therefore, limitations of the Supplemental Nutrition Assistance Program (SNAP, also known as food stamps) for low‐income Americans in combination with limited neighbourhood access to healthy food^12^ can make dietary modification difficult to impossible for people with SMI. Paradoxically, these circumstances can lead to both obesity and food insecurity in the population.^13^ The food insecurity‐obesity paradox is poignantly reflected in populations experiencing homelessness, where daily uncertainties regarding food access lead to unhealthy eating patterns that can persist even when housing is available.^14^ Food security and social justice are intrinsically related and have given rise to the concept known as food justice. Food justice has become a familiar term in the discourse on food insecurity, and it is used in a variety of diverse frameworks with different interpretations. Following the typologies of Moragues Faus, in this manuscript, we are considering food justice from a modified distributive justice frame. By distributive justice, we understand socio‐economic structures, including lack of affordable housing, as the basis of food insecurity and call for policy‐level change to increase health equity.^15^

**Figure 1:**
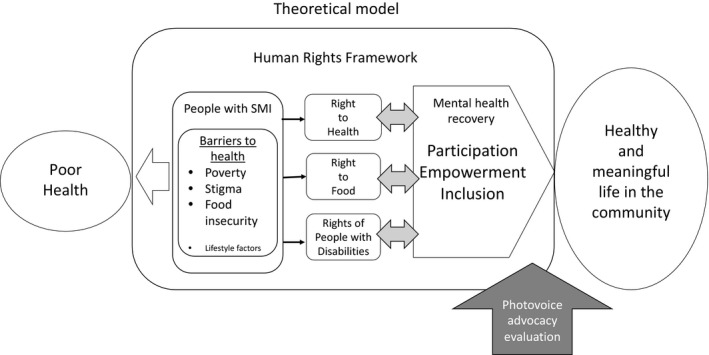
Theoretical framework, focus on advocacy

A human rights framework holds substantial potential to improve the understanding of the complexity and interrelatedness of these multilevel issues in the public and policy sectors by explicitly linking the problem of poor health in people with SMI to the right to health, the right to food and the rights of people with disabilities.^16^, ^17^, ^18^, ^19^, ^20^ There are multiple socio‐economic and policy food justice factors limiting the right to healthy food in people with SMI including limited knowledge, skills and experience with food purchasing, meal planning and preparation, and food storage, poor‐quality food in institutional settings, as well as experiences of homelessness/marginal housing, and living in areas without access to affordable, high‐quality grocery stores. Utilizing a human rights framework is an important mechanism in addressing poor health and discrimination in people with SMI because this framework (a) explicitly acknowledges the role of the social, political and economic determinants of health (SDH), (b) provides a mechanism to address these structural barriers and unequal power relationships at the local, state and national policy levels and (c) calls for a participatory response to the problem as an emancipatory practice as well as an ethical imperative.^21^, ^22^, ^23^ We acknowledge the constraints of the researcher‐driven context of this project, with further discussion below. Nevertheless, we endeavoured to situate this project in a human rights framework and use a limited participatory action research strategy to assure that the population is included in understanding and addressing the causes and consequences of poor health. An overview of the project framework is shown in Figure 1.

Inclusion, participation and empowerment are just as much a component of a human rights framework as they are elements of mental health recovery. The terms recovery and empowerment have a diverse variety of meanings for different groups, with sometimes marked tension among definitions by people with lived experience, policymakers and academic researchers.^24^ As a result, these terms have experienced a great deal of conceptual drift in a wide variety of disciplines, necessitating a high level of specificity when referring to these concepts as well as acknowledgement of the implicit assumptions in the choice of definitions.^25^ With these caveats, for this project, we are using the working definition of mental health recovery by the Substance Abuse and Mental Health Service Administration (SAMHSA) as: ‘A process of change through which individuals improve their health and wellness, live a self‐directed life, and strive to reach their full potential’.^26^ Additionally, we are referring to empowerment in a mental health context using the World Health Organization (WHO) definition; ‘the level of choice, influence and control that users of mental health services can exercise over events in their lives’.^27^ Despite these advances in policy, people with SMI are still practically disempowered at many levels including (a) the societal/structural level because of stigma, housing instability and barriers to access, (b) the organizational level from being poorly informed, treated and consulted and (c) the individual level from internalized stigma, which is linked to poor health. The public health community must find creative, meaningful and effective ways to ensure inclusion and participation. Authentic inclusion leading to active participation is one way that people with SMI can increase choice, influence and control in their lives and communities. Photovoice is one potential mechanism.^28^

### 1.1 Strategies to increase self‐advocacy and community participation

Photovoice has been used as an accessible method to amplify the experiences of marginalized populations and bring hidden social justice issues to light using photography. The Photovoice approach was first described in Wang and Burris’ seminal work^28^ and grounded in Freire's emancipatory educational methods.^29^ Photovoice methodology provides cameras to participants to identify community assets and needs, followed by critical dialogue about the meaning of these photographs in the participants’ lives. Photovoice has been applied with populations experiencing SMI,^30^, ^31^, ^32^ homelessness^33^ and people experiencing food insecurity.^21^, ^34^, ^35^, ^36^

The final goal of Photovoice is social action and political change. However, Photovoice projects have received some criticism for raising ‘false hopes’ for policy and social change as well ‘a vagueness’ in how social action plans have been described and evaluated.^37^ Johnston also notes ‘concerns for the noticeable lack of documented follow‐through actions of attempts at social change and project outcomes’.^37^ Sanson, Evans‐Agnew and Boutain^38^ explored social justice intent in Photovoice projects. Less than half of the studies included in their review described a guiding methodology. All studies reported an increase in awareness of social justice issues in participants and audiences, although few described projects that directly improved or transformed unjust conditions.^38^ Examples of successful transformations that have developed from Photovoice projects include (a) passage of a bill to strengthen accessible parking laws in the state stemming from a Photovoice project with people with spinal cord injuries^39^ and (b) enactment of a law requiring licensing of all tobacco vendors as a result of a project with Asian American and Pacific Islander youth focusing on tobacco use.^40^

We recently completed a Photovoice project^41^ to explore the barriers and facilitators to healthy living in partnership with people with SMI. These participants were part of an on‐going (June 2014‐June 2019) larger hybrid type 1 trial testing the effectiveness and examining the implementation of a 12‐month, peer‐led healthy lifestyle intervention (Peer Group Lifestyle Balance, PGLB) in supportive housing agencies serving participants with experiences of SMI and homelessness who are overweight or obese.^42^ While acknowledging the inherent limitations in situating a participatory advocacy project within a researcher defined randomized controlled trial, we felt it compulsory to critically examine the feasibility of public presentation and the participants’ experience as authentically as possible, and separately from describing the findings of the Photovoice project itself. Our hypothesis was that participation in the Photovoice process would result in community advocacy activities such as presentations and policy recommendations to influential private and public mental health organizations and these advocacy activities would support study participants in acting as change agents in their own lives. Thus, the two aims of this project were as follows: (a) to evaluate the feasibility of community advocacy resulting from the Photovoice project and (b) to explore participants’ experience of these activities, again while acknowledging the unavoidable power differential between the researchers and the study participants.

## 2 MATERIALS AND METHODS

### 2.1 Setting

Eight individuals with SMI participated in this project from April 2017‐June 2017. The project was implemented in partnership with Pathways to Housing PA, a supportive housing agency working to end homelessness for people with SMI in the city of Philadelphia.^43^, ^44^ Pathways to Housing PA uses a housing first model which offers immediate access to permanent supportive scattered‐site housing for people with experiences of chronic homelessness and SMI without preconditions.^44^ Pathways to Housing PA currently houses 300 people in individual one‐bedroom apartments throughout the city. Pathways to Housing PA has an on‐going commitment to community inclusion and policy change, making it an ideal setting for a Photovoice project.^45^, ^46^, ^47^

### 2.2 Participants/Co‐Researchers

Potential participants were recruited from those in the intervention arm of the Peer Group Lifestyle Balance study explained above.^42^ To be eligible for the Photovoice advocacy project, PGLB participants had to have completed the 12 weekly core education sessions of the PGLB curriculum at Pathways to Housing PA and Project HOME. Research coordinators for the PGLB project provided a list of the 21 eligible participants. These participants were briefly informed about the Photovoice project through the PGLB staff and given an informational flyer. All eight participants from Pathways to Housing PA expressed interest and commitment to attending the programme. Three Project HOME participants initially expressed interest but did not attend the first session despite 2 reminder calls. Therefore, a total of 8 people, all from Pathways to Housing PA, joined the Photovoice project. All eight co‐researchers participated in both the photo‐taking and discussion section of the project. In the first session of the Photovoice project, participants completed a written consent form and were introduced to the theory of participatory action research. The study protocol was approved by the institutional review boards at Thomas Jefferson University and the Philadelphia Department of Public Health with a reciprocal IRB authorization agreement from Drexel University. Participants were considered as community co‐researchers and will be referred to as such in the rest of the manuscript. All co‐researchers asked that their real names be used in the manuscript.

### 2.3 Photovoice procedure

The Photovoice procedure has been described in detail elsewhere.^43^ The following is a summary of the approach. All Photovoice sessions occurred weekly in an agency conference room and were audiotaped and transcribed. The first session was an educational session on Photovoice methodology, photography ethics and camera use. Each co‐researcher received a Vivitar ViVi Cam 9112 digital camera and participated in an on‐site practice photo‐taking session. The staff assisted the co‐researchers in downloading their pictures to a computer and projector. Co‐researchers practiced explaining their projected practice photographs to the group, using the SHOWeD format.^28^ The SHOWeD format asks co‐researchers to answer the following questions about their photograph: What do you See here? What is really Happening? How does this relate to Our lives? Why does this problem or strength exist? What can we Do about it?^28^, ^47^

After the first session, co‐researchers were given a take‐home ‘assignment’ with the following printed instructions: (a) Think about what in your environment helps or prevents you from losing weight, (b) Take as many pictures as you want, (c) Try to take pictures on both different topics: things that make it easier to lose weight and things that make it harder, and (d) Pick three to five pictures to share with the group at the next session. In weekly group sessions two through seven, we used the same initial assignment questions as a springboard to deeper discussion of identified community issues, strengths and challenges. In later sessions (five, six and seven), participants selected photographs for group presentation, identified common themes and planned for the advocacy phase of the project.

### 2.4 Procedure for advocacy presentations

All co‐researchers from the Photovoice sessions were invited to engage in the advocacy sessions preparation, presentation and post‐discussion. When the internal photograph‐taking and group discussions of the Photovoice project were complete, the university researcher contacted the leadership of the Philadelphia Department of Health Division of Chronic Disease Prevention and the co‐researchers were invited to introduce their project and explore opportunities for collaboration and participation. At this meeting, the co‐researchers presented an overview of the project using power point slides of photographs and captions that represented the major themes of the project. Each co‐researcher presented 1 to 2 slides, and all participated in the discussion with the health department staff, and the university researchers were present to support the community co‐researchers; however, the community co‐researchers were the primary presenters and discussants.

Following the initial presentation to this group, other opportunities rapidly emerged by word of mouth through the local network of agencies working for food justice. Each invitation was reviewed with the community co‐researchers and the group collectively considered each opportunity. The co‐researchers decided on the topics to cover at three of the four speaking opportunities. For the Tuesdays with Toomey opportunity, the agenda had been set by the larger organization to focus on cuts to SNAP benefits. These activities are summarized below and in Table 1.

**Table 1:** Summary of advocacy sessions

Event	Date	# PTH Part.	Description	Context	Attendees	# of attend.	Social media
PDPH planning meeting/PDPH	4/27/17	4	Co‐researchers presented an overview of the project using power point slides of photos and captions that represented the major themes of the project. Each co‐researcher presented 1 to 2 slides, and all participated in the discussion with the health department staff	Planning for Food, Fit, Philly Coalition presentation	PDPH Division of Chronic Disease Prevention Staff	6	
Good Food for All conference/Philadelphia Free library	5/11/17	4	Co‐presented with the policy director of the Coalition Against Hunger in a breakout session entitled ‘Advocacy 101’, to illustrate ‘advocacy in action’. Same presentation procedure as above	Co‐presented with policy director of the Coalition Against Hunger	People who are SNAP eligible, hunger fighters, food educators and advocates for food access	30	Twitter
Tuesdays with Toomey‐Protect SNAP /2nd and Chestnut	5/23/17	4	Co‐researchers joined in the session attended by approximately 100 people on the sidewalk outside of Senator Toomey's office and participated by holding signs, chanting, and interacting with other food justice advocacy groups. One co‐researcher gave a 4‐minute speech focused on what SNAP cuts would mean to the elderly and people with disabilities	Occurs outside Senator Toomey's offices every Tues to ask the Senator to listen and address the needs of all PA constituents	Community organizers, advocates, and members, media	approx. 100	Twitter
Food, Fit, Philly Coalition monthly meeting/ Thomas Jefferson University	6/26/17	5	Co‐researchers presented an overview of the project followed by an in‐depth review of power point slides of photos and captions that represented the major themes of the project. In addition, the researchers constructed a photography exhibit of all the photographs and captions from the project in the lobby outside the auditorium and were available to answer individual questions from the attendees following the presentation	Convene stakeholders in public health, Identify opportunities for collaboration, engagement, and action.	Get Healthy Philly, Penn Center for Public Health Initiatives, Philadelphia City Planning Commission, Health Promotion Council, Thomas Jefferson University, The Food Trust	21	

The second presentation took place at the Good Food for All Conference. Members of our Photovoice group were invited to co‐present with the policy director of the Coalition Against Hunger in a breakout session entitled ‘Advocacy 101’. The purpose of our half of the presentation was to illustrate ‘advocacy in action’. The third presentation was part of ‘Tuesdays with Toomey’ an advocacy group that met every Tuesday outside of Senator Toomey's local offices across the state to ask Senator Toomey to listen and address the needs of all Pennsylvania constituents. The Tuesdays with Toomey group was planning to focus their next Tuesday session following the presidential proposal to cut the SNAP programme by 25 per cent in the 2018 budget. Four co‐researchers joined the session advocating to protect SNAP benefits. The final presentation took place at the monthly Food, Fit, Philly Coalition. Co‐researcher presented their photographs and constructed a photography exhibit of all the photographs and captions from the project.

### 2.5 Procedure for post‐advocacy session focus groups

We held focus groups with the participants after each of the four community advocacy activities to explore their experience with public speaking, presenting their experiences and advocating. The four focus groups were semi‐structured and used the same interview guide at each session. All members of the Photovoice group were invited to these sessions, attendance ranged from three to seven participants. We opened each focus group with a review of the community activity for those that were unable to attend.

The topics for the focus groups related to concepts of human rights, mental health recovery and advocacy in people with SMI such as empowerment, participation, influence and inclusion.^48^, ^49^ These topics were designed to explore if and how the co‐researchers experienced empowerment and participation through the process of planning and presenting their photographs and captions. For this project, many of these concepts were operationalized drawing from the Equality Measurement Framework domains of participation, influence and voice, focusing on human rights^,^ ^50^ and the National Consortium on Stigma and Empowerment, focusing on mental illness.^51^ Concepts from the following measurement scales used to inform the focus group guide include: the Empowerment Scale,^52^ the Recovery Assessment Scale,^53^ the Self‐Determination Scale^54^ and the Attribution Questionnaires.^55^

### 2.6 Analysis

#### 2.6.1 Descriptive analysis

The principal university researcher documented descriptions of all community advocacy activities occurring following the first six months of the project. A summary of the advocacy sessions is shown in Table 1

#### 2.6.2 Qualitative analysis

The post‐advocacy activity focus group session recordings were transcribed verbatim using a professional transcription service and checked by the research coordinator. A research team consisting of the two university Photovoice facilitators and the university researcher who performed the transcriptions began qualitative analysis of the focus group transcripts, using a modified grounded theory approach.^56^ Nvivo 11 software^57^ (QRS International) was used to assist in organizing the qualitative analysis. We began with open and exploratory coding of the data into categories and concepts of meaning and developed a codebook. Next, we reflexively considered relationships among the codes through axial coding, and we concluded with a process of selective coding, identifying emergent themes. Throughout the process, we considered supporting and discrepant data in relation to the themes to enhance the rigour of the findings. In addition to the open coding, we also re‐coded the data using a more structured approach considering issues of empowerment, participation and non‐discrimination. We used the following strategies to enhance validity of the findings: (a) prolonged engagement and fieldwork with the population over a 6‐month period, (b) iterative member checking in a group format with the co‐researchers after multiple cycles of analysis and (c) peer review to examine the relationship between the data and conclusions.^58^, ^59^

## 3 RESULTS

### 3.1 Participant characteristics

Seven of the eight Photovoice project participants participated in at least one advocacy session. One participant was unable to join the advocacy sessions due to complications with a chronic health condition. Of the seven remaining participants, five were male and two were female. Five were Black and two were White.

### 3.2 Overview of advocacy projects

Between April and June 2017, the participants presented at 4 advocacy activities, each activity was followed by a focus group. The 4 advocacy activities included the following: (a) meeting with the director and staff from the Philadelphia Department of Public Health (PDPH) Division of Chronic Disease Prevention to give an overview of the project and begin planning for presentation at a Food, Fit, Philly Coalition meeting, (b) ‘Advocacy 101’ Co‐presentation in partnership with policy director of the Coalition Against Hunger at the Good Food for All Conference, (c) ‘Tuesdays with Toomey: Protect SNAP Benefits’ Demonstration outside Senator Toomey's office, and (d) presentation of the final project and photograph exhibition at the Food, Fit, Philly Coalition June meeting. These activities are detailed in Table 1.

### 3.3 Focus group results

#### 3.3.1 Themes

**Figure 2:**
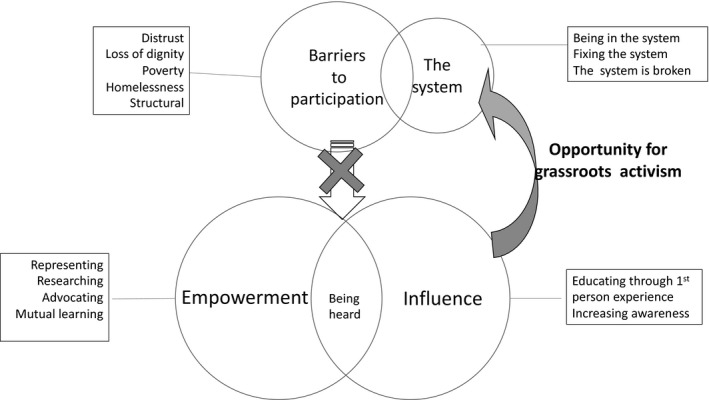
Themes from advocacy presentation debrief sessions

The focus group interview guide drew from conceptualizations of mental health empowerment, referring to the level of choice, influence and control that users of mental health services can exercise over events in their lives.^27^ Two overlapping themes emerged from the analysis: (a) Empowerment and (b) Barriers to Empowerment. The theme of empowerment arose from the subthemes of Being Heard, Advocating, Representing, Researching, Mutual Learning, Raising Awareness and Educating others through first person accounts. The System and Distrust were the two subthemes that formed the theme of Barriers to Empowerment. A diagram of the relationship between the themes and subthemes is shown in Figure 2 below and supporting quotations from co‐researchers are shown in Table 2. Ellipses are used within the quotes to represent the intentional omission of words or phrases for brevity and with the intention of not altering the original meaning. These main themes summarize the participants’ recent experience of community presentation and advocacy as well as their deep familiarity with poverty and injustice.

**Table 2:** Subthemes with supporting quotes and relationship to MH empowerment

Quotes	Subtheme	MH empowerment construct
I think the people there help a lot of school children. I think we were something different for them. Sandra	Being heard	(+) Choice: social inclusion (+) Control: contributing to the wider community
A few of them was hearing this for the first time…They didn't know it was that deep or that we was serious…And it was like uh…we was prepared for them…And then it shocked em Irwin	Being heard Educating through 1st person experiences	(+) Control: challenging unjust power relationships
Yeah, it was educational on both sides. Learning, you know, from each other Stephan	Mutual learning	(+) Choice: access to public activities
We should all take notes and research about, you know, about what we're presenting … to different groups … so we can investigate it to our fullest as we present it. Stephan	Researching	(+) Control: self‐reliance (+) Control: contributing to the wider community
You is doing all the research and you collecting all the stuff and information and everything. As far as us being at these presentations and these people, how and what do you think? We really don't know what people feel, but how do you see it, or how do you think people accepted us? Irwin	Mutual learning	(+) Choice: social inclusion (+) Control: contributing to the wider community Control: dignity and respect
I think they were influenced with the opinions of the corner stores open and those things about corner stores…You made a lot of good points, yeah, I saw it in the audience. I saw it on their faces, you know. Lois	Raising awareness Educating through 1st person experiences	(+) Influence: decision‐making process
It can promote awareness about living healthy lives, you know especially people with mental illness, you know they say you know a nut can tell a nut what to do. Anthony	Raising awareness Representing	(+) Control: contributing to the wider community
I don't think we can change their minds, but [we can] provoke thought. Anthony	Raising awareness	(+) Influence: decision‐making process
You stand in front of somebody's welfare office. Because people's comin' to get they card turned on and get some food stamps… Let um know that they ‐‐ they ain't no food stamps gonna be available no more. Irwin	Advocating	(+) Influence: social/political
I learned that some of their questions weren't just about the photo choice or our pictures, it was about our general lifestyle, and how we shop, where we go and how far, you know. But after seeing the pictures that raised more questions.	Raising awareness Representing	(+) Choice: social inclusion (+) Control: contributing to the wider community
Makes me have to go to the system's office and argue because I don't have enough…It's it's like [pause] I feel as though, at times the government be playing with you…So when I got to keep on running my bills down there…I'm just seeing that's a part of uh being in the system. Irwin	Being in the system	(−) Choice: access to information (−) Influence: decision‐making process (−) Control: dignity and respect
I mean 'cause people woke up one day and realized they weren't givin' out no more cash, they did that without, without anybody even knowing that was gonna happen… there was lines at the ATM. ‘My card's not working’. Yeah your card's not workin' 'cause youse don't get nothin' no more. Anthony	Distrust Loss of dignity	(−) Choice: access to information (−) Influence: decision‐making process (−) Control: participation in decision making
Now this the other flip side to this. He talkin' bout' doing this to the American people, having people go hungry to build a damn wall. Irwin	System is broken	(−) Influence: social/political (−) Control: dignity and respect
He's not even recognizing or realizing people are, recognizing people are standing out there doing that every Tuesday. I mean he knows what Tuesdays are gonna be, so he's not gonna show up. Anthony	Distrust	(−) Influence: social/political (−) Control: dignity and respect
Like I said before, we got a lot of people in America starving…Going through trashcans. Food is locked away in this country. Kenny	System is broken	(−) Choice: access to information (−) Control: dignity and respect
I'm just seeing that's a part of uh being in the system. But still I feel as though a lot of it's unnecessary. It's common sense that an individual must eat. Irwin	Being in the system	(−) Influence: social/political
You take a look at uh us, we was homeless at one time…we was in them lines…You understand? See people like us, we been through that, you know? But it's no fun in that. It's no dignity. It's nothing to instill in your children with that	Loss of dignity Homelessness	(−) Control: dignity and respect
There's a lot of out of sight out of mind… You know what I mean? So then obviously they don't know… Now I got 8 beer distributors in my neighborhood, right?	The system	(−) Influence: decision‐making process (−) Control: challenging unjust power relationships

#### 3.3.2 Being heard

Several participants remarked that they brought a new group of voices to the topic of food justice, leading to the subthemes of Being Heard. For example, when reflecting on the discussion with the staff at the Philadelphia Department of Health Division of Chronic Disease Prevention, Sandra explained, ‘I think the people there help a lot of school children. I think we were something different for them’. Irwin commented on the experience at the Good Food for All Conference presentation, ‘A few of them was hearing this for the first time…They didn't know it was that deep or that we was serious…And it was like uh…we was prepared for them…And then it shocked em’. Expanding on the idea of being heard, participants also appreciated the opportunity for mutual learning. Again, reflecting on the PDPH planning meeting, Stephan explained: *‘*Yeah, it was educational on both sides. Learning, you know, from each other’. Additionally, participants spoke with pride in representing their agency: ‘I was happy that I was there to speak on Pathways' behalf as far as, and our nutrition group you know what I mean?’

Reflecting on a different facet of empowerment, participants spontaneously critiqued their role as community researchers and advocates, as Stephan explained, ‘We should all take notes and research about, you know, what we're presenting to the environment and to different groups and you know so it can be more investigative, or we can investigate it to our fullest as we present it’. Participants were also very interested to receive feedback on their presentations and discuss the opportunity for Mutual Learning, as Irwin asked, *‘*You is doing all the research and you collecting all the stuff and information and everything. As far as us being at these presentations and these people, how and what do you think? We really don't know what people feel, but how do you see it, or how do you think people accepted us?’

The subtheme of Raising Awareness complemented the themes of Empowerment and Educating Through 1st Person Experiences. Lois spoke positively about the presentation by Anthony at the Food, Fit, Philly Coalition meeting: ‘I think they were influenced with the opinions of the corner stores open and those things about corner stores…You made a lot of good points, yeah, I saw it in the audience. I saw it on their faces, you know’. More specific to the participants’ experience, Anthony also noted, ‘it can promote awareness about living healthy lives, you know especially people with mental illness, you know they say you know a nut can tell a nut what to do’. At the same time, participants were realistic about their influence in the policy realm, as Anthony expressed: ‘I don't think we can change their minds, but [we can] provoke thought.’ and Stephan responded, ‘Make them more aware’.

Civic engagement is an important element of raising awareness. The participants demonstrated increasing awareness of local issues, particularly after participating in a weekly ‘Tuesday's with Toomey’ event (these events occur outside Senator Toomey's offices every Tuesday to ask the Senator to listen and address the needs of all PA constituents). For example, a few weeks after participating in the demonstration at Senator Toomey's office, Anthony shared: *‘*Now see Toomey's back in the uh ‐‐ back in the news again today… they had a protest yesterday in front of his office. Everybody in wheelchairs… it was pretty big. It actually made the news this morning’. After taking part in the demonstration, participants voiced other ways to catalyse change by raising awareness: ‘You stand in front of somebody's welfare office. Because people's comin' to get they card turned on and get some food stamps… Let um know that they ‐‐ they ain't no food stamps gonna be available no more’. (Irwin) In response, Anthony affirmed: *‘*Yeah and the word'll get out’.

The participants recognized the usefulness of the Photovoice process as a method to educate and influence the public on real‐life socio‐economic food justice issues faced by people living in areas with limited access to healthy foods. Reflecting on their presentation at the Food Fit Philly meeting, Stephan commented, *‘*I learned that some of their questions weren't just about the photo choice or our pictures, it was about our general lifestyle, and how we shop, where we go and how far, you know. But after seeing the pictures that raised more questions’.

#### 3.3.3 Barriers to empowerment

Participants spoke directly and indirectly about the difficulty of asserting power and influence in the system and the limits to affecting policy change. Irwin gave examples of his own struggles, Makes me have to go to the system's office and argue because I don't have enough…It's like [pause] I feel as though, at times the government be playing with you…So when I got to keep on running my bills down there…I'm just seeing that's a part of, uh, being in the system. Other participants spoke about the indignities and predicaments of being in the system and not having control, *‘*I mean 'cause people woke up one day and realized they weren't givin' out no more cash. They did that without anybody even knowing that was gonna happen… there was lines at the ATM. ‘My card's not working’. Yeah, your card's not workin' 'cause youse don't get nothin' no more’ (Anthony).

Participants also noted the inconsistencies and lack of response from national policymakers. Irwin explains, ‘Now this, the other flip side to this. He talkin' bout' doing this to the American people, having people go hungry to build a damn wall’. Anthony recognized this same attitude from US Senators, ‘He's not even recognizing or realizing people are, recognizing people are standing out there doing that every Tuesday. I mean he knows what Tuesdays are gonna be, so he's not gonna show up’. (Anthony) Correspondingly, participants appreciated the various ways that food insecurity presents in US society, it ‘is really a serious matter, you know what I mean? This is not just a Pathways' thing…Or a homeless thing…It's a nationwide serious problem and something really needs to be done about it’ (Irwin).

In addition, participants spoke of a lack of understanding on the part of decision makers, as well as their ineffectiveness, ‘It's a lot of different avenues, but it seemed like with all the different avenues combined together it's still don't work. The system is still broke. That's what I learned’. (Irwin) Participants also focused down to the issue of food injustice in the system, again, as Irwin remarked: ‘I'm just seeing that's a part of being in the system. But still I feel as though a lot of it's unnecessary. It's common sense that an individual must eat’.

The system, referring to city, state and national social service policies, as the major multilevel barrier voiced by the participants. However, additional important barriers to participation framed the discussion. Co‐researchers often express general distrust the system, as Irwin explained, ‘Why? Because we have no other means, and it's the government. And we supposed to be the greatest nation, and we starving? What kinda shit is that? …that means that the government is doin' this to us, so how can we trust or listen to the government any longer?’ This distrust is compounded by experiences of indignity and food insecurity during periods of homelessness and extreme poverty, ‘You take a look at uh us, we was homeless at one time…we was in them lines…You understand? See people like us, we been through that, you know? But it's no fun in that. It's no dignity. It's nothing to instill in your children with that. (Irwin)’ Living in poor neighbourhoods with easy access to alcohol and illegal drugs affected the participant‐researchers both personally, and on the community level. Anthony articulated his opinion that people from the suburbs do not realize the barriers they face in the inner city, *‘*There's a lot of ‘out of sight out of mind’… You know what I mean? So then obviously they don't know… Now I got 8 beer distributors in my neighborhood, right?’ Co‐researchers even discussed the unintended consequences of policies designed to improve health and quality of life, for example the recent Philadelphia city tax imposed on sugary drinks. Again, Anthony explained the situation in the city as opposed to the suburbs, ‘They're trying to stop a reversal of it, but see … it's only hurting the in the inner city. It has no effect in the suburbs… I consider it a real treat to get a soda nowadays’. Anthony's response to the facilitator's question ‘Could that [cutting down on soda] be a good thing?’ revealed a truth often not recognized by policymakers. ‘No, actually it's not because I consume more beer, I mean, I don't have $2 for a for a bottle of soda, I can get two beers for that’.

For the most part, the presentations were a very positive experience for the co‐researchers. We asked the participants specifically about several disability‐related barriers after every presentation. None of the participants endorsed any experience of stigma or judgement during the presentations, and there was only the occasional structural barrier mentioned in terms of long travel times on public transportation. However, when asked about the audiences’ perceptions, some comments revealed long‐standing internalized stigma regarding mental illness. For example, when asked, ‘Have you guys felt judged before, in other public circumstances?’ Anthony remarked, ‘When I don't take my medication, I feel that way’. Reflecting familiarities with discrimination, some participants urged caution when participating in demonstrations such as Tuesdays with Toomey. As Irwin explained, *‘*You understand if it calls for that then it calls for that, but you also have to have people that's gonna bail your butt out… You cannot have a criminal record, or anything of that nature. You know what I mean? Or wanted or anything like that…You ain't comin' outta jail’. Comments such as these reflect a pragmatic response to the realities of racism towards African Americans and classism experienced by the co‐researchers.

## 4 DISCUSSION

The purpose of this analysis was not only to document the feasibility of advocacy activities arising from a Photovoice project, but also to explore the experience of the participants during these activities. This evaluation adds to the evidence that with people with experiences that contribute to food insecurity, such as homelessness and SMI, want to participate in food justice advocacy activities, such as presentations and discussions with local policymakers, and are met with interest and acceptance by a wide range of audiences.^28^, ^32^ Data from the focus groups confirm that the co‐researchers saw themselves as active participants in society and provided emergent evidence of choice, influence and control in their everyday lives as individuals and in community, as well as barriers to exercising these powers. We reach this conclusion through the diversity and reach of the advocacy activities and through the structured conversations with the participants reflecting strength and insight into the concept of mental health empowerment. While these were only four activities, and far less than those of other larger photovoice programmes such as Witnesses to Hunger,^21^ the success of our project further supports the inclusion of people with experiences of SMI and homelessness in advocacy. The focus on systems as problematic and potentially oppressive is also in synch with the Witnesses to Hunger photovoice study, as well as with others that identify serious problems with participant experiences with public assistance programming.^21^, ^36^

The human rights framework provides a useful lens to review the advocacy activities themselves as well as the co‐researchers experiences with the activities. The *Good Food for All Conference* and the *Tuesdays with Toomey* event provided an opportunity for the co‐researchers to directly advocate for the right to health, the right to food and the rights of people with disabilities.^17^, ^18^, ^19^, ^20^, ^60^ During the *Tuesdays with Toomey* event, one co‐researcher volunteered to embody the rights of people with disabilities through his presentation and call to action during the demonstration. During follow‐up discussions, the co‐researchers identified many threats to their right to health and healthy food as reflected in discussions of food insecurity, lack of healthy food and easy availability of alcohol in corner stores, cuts to SNAP and welfare benefits, and the reality of food stamp fraud. Discussions around the topics of stigma, distrust and indignity reflected barriers to the rights of people with disabilities. The presentations to the staff at the Philadelphia Department of Public Health as well as the Food Fit Philly Coalition meeting provided the co‐researchers with the opportunity for participation and advocacy through discussions with policymakers and government officials. In follow‐up discussions from these sessions, the co‐researchers identified experiences and perceptions that contribute to mental health recovery such as being heard, not being judged, mutual learning and influencing policymakers.

Exclusion, discrimination and oppression are commonplace experiences for people with psychiatric disability.^61^, ^62^, ^63^ This project provides evidence that a simple process such as giving people a camera to document and discuss their experiences in a supportive environment can provide marginalized groups with a means to raise awareness about hidden barriers to health and well‐being as well as amplify their own voice, strength and self‐determination and contribute to their recovery. Papoulias highlights the potential of Photovoice in translatability of health‐care improvement research and asserts ‘photography becomes a means of translating local concerns into a community “voice” which in turn becomes legible to a wider audience of policymakers and clinicians’.^64^ The experiences of our group suggest the preliminary emergence of a community voice. At the same time, we recognize the limitations of directly addressing distributive justice and agree with recent recommendations that Photovoice be regarded as a tool for mental health policy informing rather than policy changing.^37^ As Anthony insightfully remarked, ‘I don't think we can change their minds but provoke thought’. Nevertheless, our evaluation confirms participants’ felt the project to be a worthwhile experience, as Stephan summarized, ‘yea it was educational on both sides. Learning, you know, from each other’.

### 4.1 Limitations

The co‐researchers were limited in their participation a priori as a consequence of situating this project in a larger federally funded randomized controlled trial.^42^ As noted in the introduction, this context also constrained the definition and experience of recovery and empowerment to a more conventional and researcher‐based agenda.

The small number of participants in our project functioned well in a group setting and could articulate their views and experiences. In this way, they may not be reflective of the entire cohort of 314 participants in the parent project. Photovoice projects have been criticized for not adequately evaluating their impacts on the policy level.^45^ This is understandably attributed to the complex and long‐term nature of policy change. Our own project is certainly limited in this aspect as well. However, the Photovoice group was satisfied to see that a few months after they attended the demonstration, Senator. Toomey did agree to meet with constituents at an in‐person town hall for the first time in months.

The principal university investigator for this Photovoice project was involved with the larger project on several levels. She is a co‐investigator on the parent project and supervises two of the peer specialists who deliver the intervention. This researcher is also the Director of Integrated Care at Pathways to Housing, and she serves as the primary care physician for a majority of co‐researchers in the programme, including some of the co‐researchers in this Photovoice project. Clearly, there is a significant power differential between the university researcher and her co‐researchers, leading to inevitably to limitations in the nature of participant responses based on perceived social desirability.^65^ Indeed, this power differential may increase the likelihood of consenting to participant in the project. Additionally, throughout the project, the university researchers medical and public health orientation and framework contributed to the interpretation of the findings.

With these limitations in mind, explicit efforts were made to equalize the creation of knowledge among the university and community researchers, for example the facilitators present during the group sessions were involved primarily in giving directions, clarifying goals of the sessions and intervening to allow all group members equal time to present their photographs. In later sessions, some co‐researchers took the lead in facilitating the discussions and eliciting input from all members. Additionally, the initial manuscript resulting from the Photovoice project was written and reviewed in partnership with the community researchers.^41^ Indeed, Kramer‐Roy^66^ advocates specifically for health‐care professionals’ involvement in creative and participatory research projects with disabled and marginalized populations. She asserts, ‘making the congruence between their profession and these methods explicit has the potential to enhance the emancipatory role of their profession as well as enabling them to use their professional skills to carry out the research well’.^66^

On an individual level, the principal university investigator acknowledges the significant benefit she has received from this project in terms of clinical growth and understanding, as well as academic advancement. While the case can be made of mutual learning between academic and community researchers, the power differential inherently limits the evidence for this assertion.

## 5 CONCLUSION

We have demonstrated additional evidence that creative and participatory methods, such as Photovoice, are an important approach for helping people living with serious psychiatric disabilities and multiple disadvantages to confidently advocate for the right to health and the right to healthy food. However, this is only a small step; larger, coordinated efforts are needed to truly co‐create the conditions for a healthy life in the community for all. Important next steps are to authentically include people with lived experience of SMI in all levels of decision making, development and funding in areas such as (a) efforts to assure access to high quality, integrated medical and behavioural health care including support for wellness services, (b) useful research that will change practice and improve outcomes in the health care of people with SMI and (c) inclusive initiatives and policies to increase affordable healthy food availability and safe places for physical activity in low‐income neighbourhoods.

## CONFLICT OF INTEREST

We confirm that all of the authors report no conflicts of interest.

## Data Availability

Data available on request due to privacy/ethical restrictions.
